# Long Non-Coding RNA MALAT1 Mediates Transforming Growth Factor Beta1-Induced Epithelial-Mesenchymal Transition of Retinal Pigment Epithelial Cells

**DOI:** 10.1371/journal.pone.0152687

**Published:** 2016-03-28

**Authors:** Shuai Yang, Haipei Yao, Min Li, Hui Li, Fang Wang

**Affiliations:** Department of Ophthalmology, Shanghai Tenth People’s Hospital, Tongji University School of Medicine, Shanghai, 200072, China; National Eye Institute, UNITED STATES

## Abstract

**Purpose:**

To study the role of long non-coding RNA (lncRNA) MALAT1 in transforming growth factor beta 1 (TGF-β1)-induced epithelial-mesenchymal transition (EMT) of retinal pigment epithelial (RPE) cells.

**Methods:**

ARPE-19 cells were cultured and exposed to TGF-β1. The EMT of APRE-19 cells is confirmed by morphological change, as well as the increased expression of alpha-smooth muscle actin (αSMA) and fibronectin, and the down-regulation of E-cadherin and Zona occludin-1(ZO-1) at both mRNA and protein levels. The expression of lncRNA MALAT1 in RPE cells were detected by quantitative real-time PCR. Knockdown of MALAT1 was achieved by transfecting a small interfering RNA (SiRNA). The effect of inhibition of MALAT1 on EMT, migration, proliferation, and TGFβ signalings were observed. MALAT1 expression was also detected in primary RPE cells incubated with proliferative vitreoretinopathy (PVR) vitreous samples.

**Results:**

The expression of MALAT1 is significantly increased in RPE cells incubated with TGFβ1. MALAT1 silencing attenuates TGFβ1-induced EMT, migration, and proliferation of RPE cells, at least partially through activating Smad2/3 signaling. MALAT1 is also significantly increased in primary RPE cells incubated with PVR vitreous samples.

**Conclusion:**

LncRNA MALAT1 is involved in TGFβ1-induced EMT of human RPE cells and provides new understandings for the pathogenesis of PVR.

## Introduction

Proliferative vitreoretinopathy (PVR), a severe blinding disease characterized by the formation of epiretinal membranes through a defective wound repair process, occurs as a complication of rhegmatogenous retinal detachment [1,2]. PVR is the primary reason for failure of initially successful retinal re-attachment surgery due to the recurrent preretinal or epiretinal membrane traction, which further leads to retinal redetachment and dramatic visual loss [3]. Several cell types are involved in the pathogenesis of PVR, including retinal pigment epithelial (RPE) cells, fibroblasts (primarily derived from RPE cells), glial cells, and inflammatory cells [4]. In all these cell types, RPE cells is thought to play the principal role in the pathogenesis of PVR as it is recognized as the largest cellular component of the epiretinal membranes in PVR patients [5]. In the settings of PVR development, RPE cells which exposed to the vitreous (which is rich of cytokines and growth factors) are detached from Bruch’s membrane and migrate into the vitreous through the retina tear [6]. In this process, RPE cells undergo a process known as epithelial-mesenchymal transition (EMT), an orchestrated series of events in which fully differentiated epithelial cells undergo transition and acquire a mesenchymal phenotype. Afterwards, RPE cells gradually participate in the formation of fibrotic membrane on the retina. These membrane contracts under the stimulation of growth factors/cytokines in the vitreous, and further leads to traction retinal detachment [7]. Therefore, fully understanding of the mechanisms of EMT in RPE cells is required for identifying potential therapeutic targets in treating PVR. Currently, an increasing number of studies were subjected to explore the mechanism and the treatment of EMT in RPE cells. ARPE-19 cells, a human RPE cell line, is frequently used to establish the EMT model because it simuates the EMT process of RPE cells solidly and sonsitantly, though it lacks some of RPE features [7–15]. Currently, various growth factors/cytokines, intracellular signaling pathways, transcription factors, and microRNAs are indicated to play significant roles in EMT of RPE cells [7–12,16]. However, it is not clear whether long non-coding RNAs (LncRNAs) contribute to EMT of RPE cells.

LncRNAs are defined as a class of non-protein-coding RNAs that are longer than 200 nucleotides in length. It was identified that lncRNAs regulates a lot of physiological or pathological processes like angiogenesis, immune response, inflammation, cell motility, and tumorigenesis. The significant role of lncRNAs in triggering EMT has also been observed in tumor metastasis [17,18]. Metastasis Associated Lung Adenocarcinoma Transcript 1 (MALAT1) is one of the best-characterized lncRNAs with multiple functions, including triggering EMT in tumor cells and promoting tumor metastasis [19,20]. Thus, we questioned whether MALAT1 contributes to EMT in fibrotic diseases, like PVR. In this study, we aimed at exploring the role of MALAT1 in EMT of RPE cells, a hallmark in the pathogenesis of PVR.

## Materials and Methods

### Reagents and antibodies

Mouse anti-human E-Cadherin antibody (for western blot) was purchased from BD Bioscience (San Jose, CA, USA). Rabbit anti-human ZO-1, Mouse anti-human α-SMA, FITC-conjugated anti mouse, and FITC-conjugated anti Rabbit antibodies were obtained from Invitrogen (Carlsbad, CA, USA). Rabbit anti-human ZEB1 antibody, Mouse anti-human fibronectin antibody was from Sigma-Aldrich (St. Louis, MO, USA). Rabbit anti-human SLUG antibody, Rabbit anti-human E-Cadherin antibody (for immunofluorescence) and rabbit anti-human β-actin antibody was purchased from Abcam (Cambridge, MA, USA). Rabbit anti-human Snail antibody was from Santa-Cruze Biotechnology (Santa Cruz, CA, USA). Mouse anti-human Smad2/3, p-Smad2/3, p38, and p-p38 antibodies were from Cell Signaling Technology (Danvers, MA, USA). TRIzol reagent and DAPI (4’,6-diamidino-2-phenylindole) were bought from Invitrogen (Carlsbad, CA, USA). SuperReal PreMix Plus (SYBR Green) reagent kit was from Takara Clontech (Kyoto, Japan). Most of other reagents such as salt and buffer components were analytical grade and obtained from Sigma-Aldrich (St. Louis, MO, USA).

### Ethics Statement

The current research involving human participants has been approved by the ethic committee of Shanghai Tenth People’s Hospital with written consents, and was in compliance with the Declaration of Helsinki. All subjects gave their written approval and consent.

Donors’ eyes were obtained from the Eye Bank of Shanghai Tenth People’s Hospital and the vitreous were collected. PVR vitreous samples were collected from PVR patients (n = 4) underwent a vitrectomy. Vitreous samples were stored at -80°C.

### Cell culture

Human RPE cell line ARPE-19 cells in our lab[8,10,21] were used in this study. the Eye Institute of Tongji University. Human primary RPE cells were isolated from donors’ eyes (obtained from the Eye Bank of Shanghai Tenth People’s Hospital) and the low passage cells (passage 2–4) were used in this study.

ARPE-19 cells and human primary RPE were cultured in DMEM/F12 culture media (Gibco, Life Technologies) with 10% FBS (Gibco, Life Technologies) at 37°C in a humidified incubator containing 5% CO_2_. The culture media was change every 2–3 days. For further experiments, cells were trypsinized and seeded in 6 or 12- well plates and cultured for 12 hours. Thereafter, cells were starved for 16 hours and were stimulated with 10ng/ml TGFβ1 (Invitrogen, Life Technologies) or vitreous samples for various time periods. In some experiments, SiRNA transfection of ARPE-19 cells was performed using the Lipofectamin ® 3000 reagent (Invitrogen, Life Technologies) according to the manufacturer’s instructions. The MALAT1 siRNA sequence (Sense: 5’-3’: GGCAAUGUUUUACACUAUUTT; Anti-sense: 5’-3’: AAUAGUGUAAAACAUUGCCTA), and the negative control siRNA sequence (Sense: 5’-3’: UUCUCCGAACGUGUCACGUTT; Anti-sense: 5’-3’ACGUGACACGUUCGGAGAATT) were synthesized by Genepharma (Shanghai, China).

### Real-time quantitative PCR

Total RNAs were extracted at indicated times with a TRIzol reagent kit. The cDNA was prepared using the PrimeScript™ RT reagent Kit (Takara Clontech, Kyoto, Japan). Real-time PCR was performed in triplicates using SuperReal PreMix Plus (SYBR Green) kit (Takara Clontech, Kyoto, Japan) on an CFX Connect Real-Time System (Bio-rad, CA, USA). Each reaction contained 12.5 μl of 2×SYBR® Premix Ex Taq™ (with SYBR Green I), 300 nM oligonucleotide primers (S1 Table) synthesized by Generay Corp., (Nanjing, China), and 1 μl cDNA in a final volume of 25 μl. The thermal cycling conditions included an initial denaturation step at 95°C for 30 s, 40 cycles of 95°C for 5 s and 60°C for 30 s. The RNA expression was normalized to the level of β-actin mRNA.

### Western blot analysis

After treatment, ARPE-19 cells were lysed in RIPA buffer (Beyotime, Shanghai, China) supplemented with phenylmethylsulfonyl fluoride and PhoSTOP EASY pack phosphatase inhibitor (Roche, Mannheim, Germany) on ice for 30 min. The lysate were clarified by centrifugation at 12000 rpm for 5 min. Total protein concentration was quantified by a bicinchoninic acid assay kit (Pierce, Rockford, IL, USA). 50 μg protein was loaded and separated on 6% and 10% SDS-PAGE gels and transferred onto nitrocellulose membrane (Bio-rad, CA, USA). To avoid non-specific binding, the membranes were blocked using 5% bovine serum albumin (BSA, Sigma Aldrich, MO, USA) in PBS for 45 min at room temperature. The membranes were then incubated with primary antibodies diluted in 2% BSA in PBS with 0.1% Tween-20 (PBS-T) at 4°C overnight. After rinsing with PBS-T for three times, the membranes were incubated with IRDye® 680LT Goat anti-Rabbit or IRDye® 800CW Goat anti-Mouse secondary antibodies (Li Cor Biosciences, NE, USA) at room temperature for 1 h. After washed with PBS-T for three times, the bound antibody was detected by Odyssey infrared imaging system (Li Cor Biosciences, NE, USA). The band intensities are analyzed to with the Odyssey software and normalized to β-actin or GAPDH.

### Immunofluorescence analysis

ARPE-19 cells were seeded and cultured in a 24-well plate inlaid with glass coverslips. After the transfection with siRNA and the treatment of TGFβ, cells were washed and fixed in cold acetone for 5 min. After washing with PBS 3 times, cells were blocked with 2% BSA for 1 h at room temperature and incubated with the primary antibodies overnight at 4°C. After rinsed with PBS 3 times, the coverslips were further incubated with FITC-conjugated secondary antibodies for 1 hour at room temperature. After counterstained with 4,6-diamidino-2-phenylindole (DAPI), the stained coverslips were mounted and visualized under a confocal microscope (Carl Zeiss, LSM710, Jena, Germany).

### Transwell migration assay

ARPE-19 cells transfected with MALAT1 SiRNA or negative control SiRNA were incubated with 10ng/ml TGF-β1 for 48 h. Then the cells were trypsinized and 5*10^4^ cells were seeded in the upper chamber of the 24-well transwell plates (8mm pore size, Costar, Conning, CA, USA) in 100 μl DMEM/F12 containing 0.5% FBS. The lower compartment were filled with 600 μl DMEM/F12 containing 10% FBS. The chambers were then incubated at 37°C for 18 h. After removing the cells on the upper surface of the filter, migrated cells on the lower surface of the culture inserts were fixed with methanol and stained with 0.1% crystal violet for 30 mins. The number of migrated cells in each chamber was then determined by counting five random fields. All the experiments were performed in triplicate.

### Scratch assay

ARPE-19 cells were seeded in 6-well plates and were transfected with MALAT1 SiRNA or NC SiRNA. After 6 h, scratches were made to the cell monolayers with a 200 μl pipette tip. Then the cells were washed twice with PBS to remove the floating cells. Thereafter, fresh serum-free medium supplemented with 10ng/ml TGF-β1 and 10μg/ml mitomycin (Sigma Aldrich, MO, USA) was added to the cells. Photographs were taken after 0, 24, and 48 h after the scratch. The cell-free area was measured and normalized to day 0 using imageJ software (V1.45 NIH, Bethesda, MD, USA). All the experiments were performed in triplicate.

### Proliferation assay

ARPE-19 cells were seeded in 96-well plates at a 2000 cells per well. After starved for 16 hours, transfections of siRNAs were performed and TGF-β1 or vehicle was added to the medium. Each experiment was performed in ten independent wells. After 48 hours, cell proliferation was assessed using the CellTiter 96 Aqueous One Solution Cell Proliferation Assay kit (Promega, Madison, WI, USA) according to the manufacturer’ s instructions. The absorbance at 490nm was quantified using a microplate spectrophotometer (Thermo, Waltham, MA, USA).

### Statistical analysis

All experiments were performed at least 3 times. The means and SEM were calculated on all parameters determined in this study. Data were analyzed statistically using one-way ANOVA or two tailed Student’s t test. A value of *P*< 0.05 was accepted as statistically significant.

## Results

### TGF-β1 induces EMT and MALAT1 expression in ARPE-19 cells

**Fig 1:**
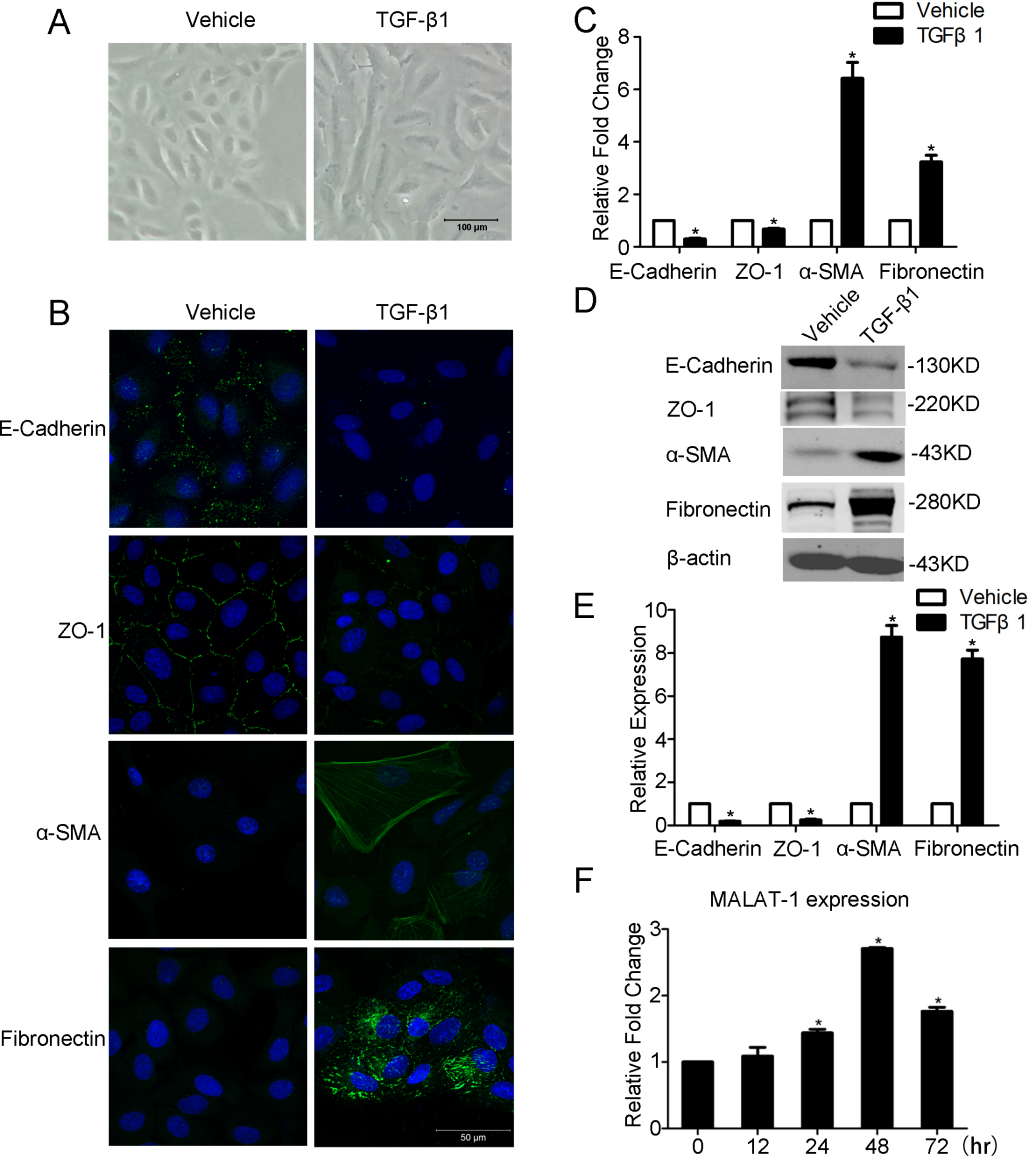
TGF-β1 induces EMT and MALAT1 expression in ARPE-19 cells. APRE-19 cells were incubated with TGF-β1 (10 ng/ml) for 48h. A. TGF-β1 induces the morphological change of APRE-19 cells were captured at 100× magnification. B-D. The expression level of EMT-related markers (E-Cadherin, ZO-1, α-SMA, and Fibronection) were detected by immunofluorescence, RT-PCR, and Western blot. E. Quantification of the relative protein expression (normalized to β-actin) in western blot. F. The expression of MALAT1 was detected by RT-PCR in indicated time points.

To decipher whether MALAT1 is involved in TGF-β1 induced EMT of ARPE-19 cells, we first treated RPE cells with TGF-β1 as previously described [8,10,21]. After incubated with TGF-β1 for 48 h, the ARPE-19 cells undergoes an EMT transition, as confirmed by its morphological change to a spindle-shaped cells, decreased expression of epithelial markers E-Cadherin and ZO-1, as well as enhanced expression of mesenchymal markers, including fibronectin and αSMA(Fig 1A–1E). We then detected MALAT1 expression in TGF-β1 stimulated APRE-19 cells at different time intervals. It was found that MALAT1 expression is gradually increased in TGF-β1 treated cells, and reaches an apex at 48 h (Fig 1F). The up-regulation of MALAT1 indicates it may contribute to EMT of RPE cells.

### Knockdown of MALAT1 attenuates the TGF-β1 induced EMT in ARPE-9 cells

We then subjected to explore the role of MALAT1 in TGF-β1 induced EMT in RPE cells by knocking down MALAT1. As the TGF-β1 induced upregulation of MALAT1 reaches the peak at 48 h, we chose this time point to examine the function of MALAT1 in the following experiments.

**Fig 2:**
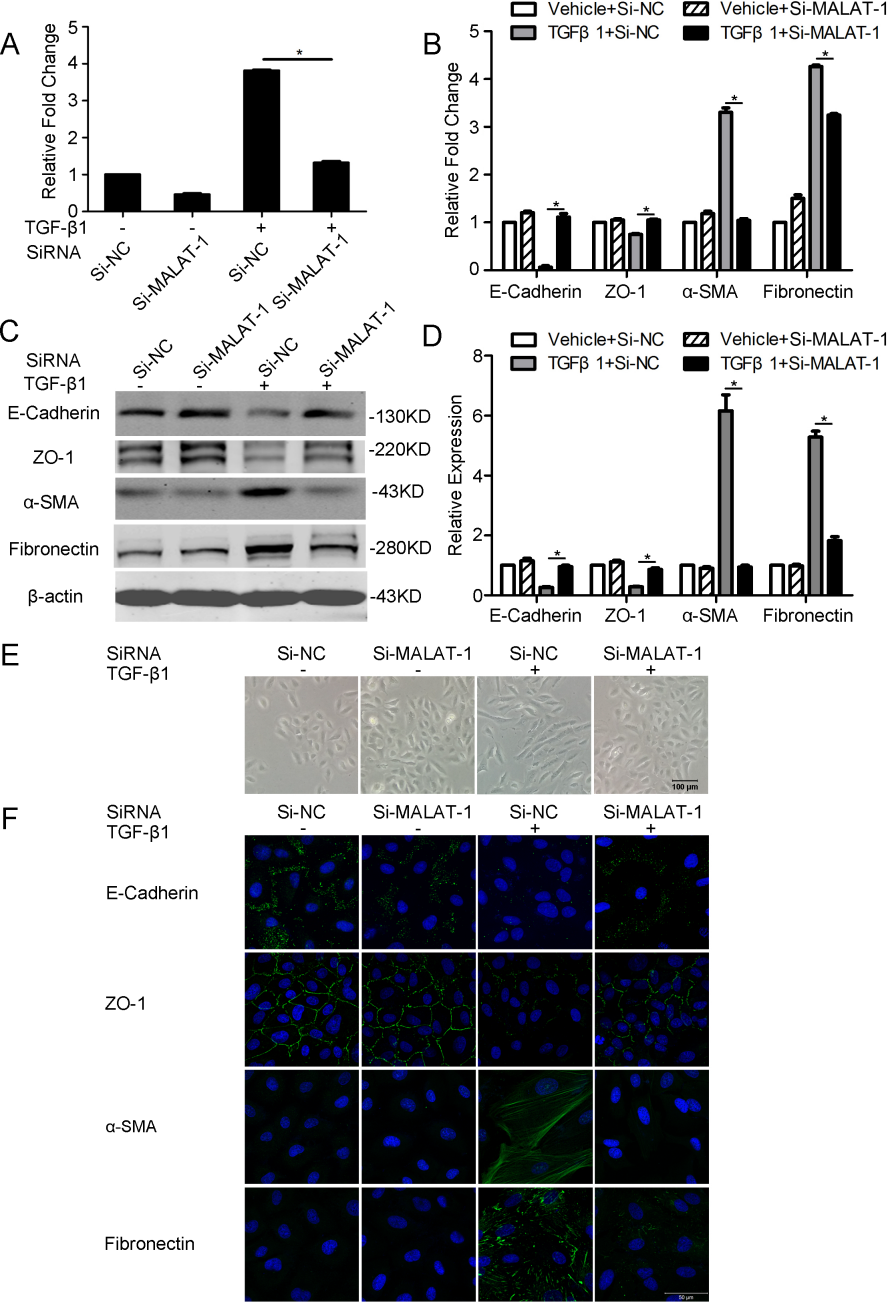
Knockdown of MALAT1 attenuates the TGF-β1 induced EMT in RPE cells. ARPE-19 cells were transfected with MALAT1 SiRNA (Si-MALAT1) or negative control SiRNA (Si-NC) and were treated with or without TGF-β1 (10ng/ml) for 48 h. A. The expression levels of MALAT1 were detected by RT-PCR. B-C. The expression level of EMT-related markers (E-Cadherin, ZO-1, α-SMA, and Fibronection) were detected by RT-PCR and Western blot. D, Quantification of the relative protein expression (normalized to β-actin) in C. E. The morphologic appearances of the cells were captured at 100× magnification. F. The expression of E-Cadherin, ZO-1, α-SMA, and Fibronection werer detected by immunofluorescence.

ARPE-19 cells were transfected with MALAT1-specific siRNA (Si-MALAT1) or a negative control siRNA (Si-NC) after starved for 16 h. After incubating the cells with TGF-β1 for 48 hours, the expression of MALAT1 was then detected by RT-PCR. Compared with Si-NC, transfection with Si-MALAT1 decreased the expression of MALAT1 by more than 60% (Fig 2A). We then subjected to detect the expression of EMT related genes including E-Cadherin, ZO-1, fibronectin, and α-SMA by RT-PCR, Western blot and immunofluorescence. It was found that knockdown of MALAT1 significantly attenuates TGF-β1 induced down-regulation of E-Cadherin and ZO-1, and up-regulation of fibronectin and α-SMA (Fig 2B–2D and 2F). Furthermore, knockdown of MALAT1 abrogates the TGF-β1 induced morphological change of RPE cells (Fig 2E). These results indicated that MALAT1 contributes to the TGF-β1-induced EMT of RPE cells.

### Knocking-down of MALAT1 reduces the TGF-β1-induced up-regulation of EMT-related transcription factors

**Fig 3:**
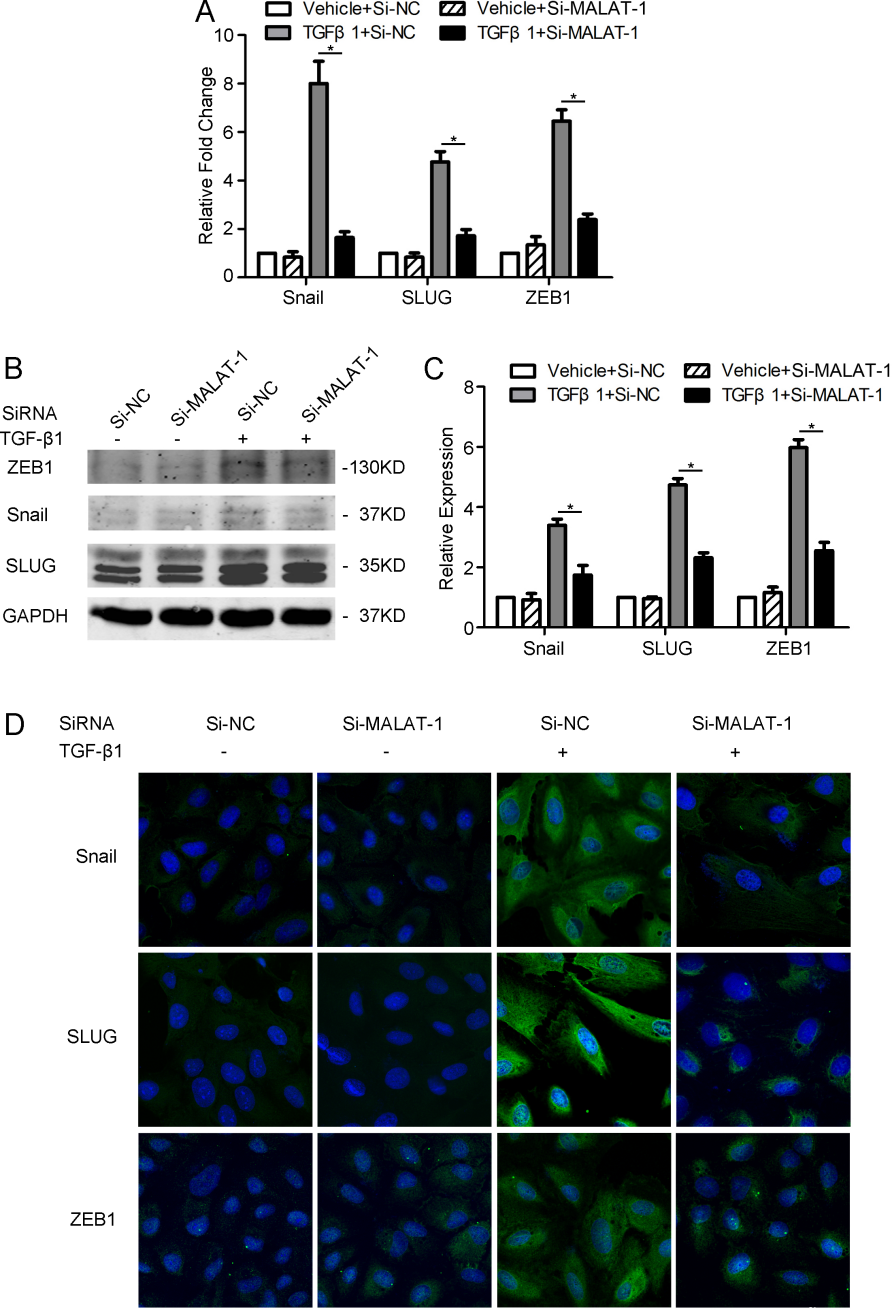
Knocking-down of MALAT1 reduced the TGF-β1-induced up-regulation of Snail, SLUG, and ZEB1 in RPE cells. **A-D** ARPE-19 cells were transfected with MALAT1 SiRNA (Si-MALAT1) or negative control SiRNA (Si-NC) and were treated with or without TGF-β1 (10ng/ml) for 48 h. The expression level of EMT-related transcription factors (Snail, SLUG, and ZEB1) were detected and quantified by RT-PCR (A), Western blot (B-C) and immunofluorescence (D).

To further confirm the role of MALAT1 in mediating the TGF-β1-induced EMT of RPE cells, we detected the expression of EMT-related transcription factors, including Snail, SLUG, and ZEB1 in ARPE-19 cells by RT-PCR, Western blot, and immunofluorescence. It was found that knocking down of MALAT-1 considerably attenuates the TGF-β1-induced up-regulation of Snail, SLUG, and ZEB1 (Fig 3A–3D). These findings reinforced the fact that MALAT1 plays a critical role in TGF-β1 induced EMT in RPE cells.

### Downregulation of MALAT1 inhibits the migration and proliferation of RPE cells

**Fig 4:**
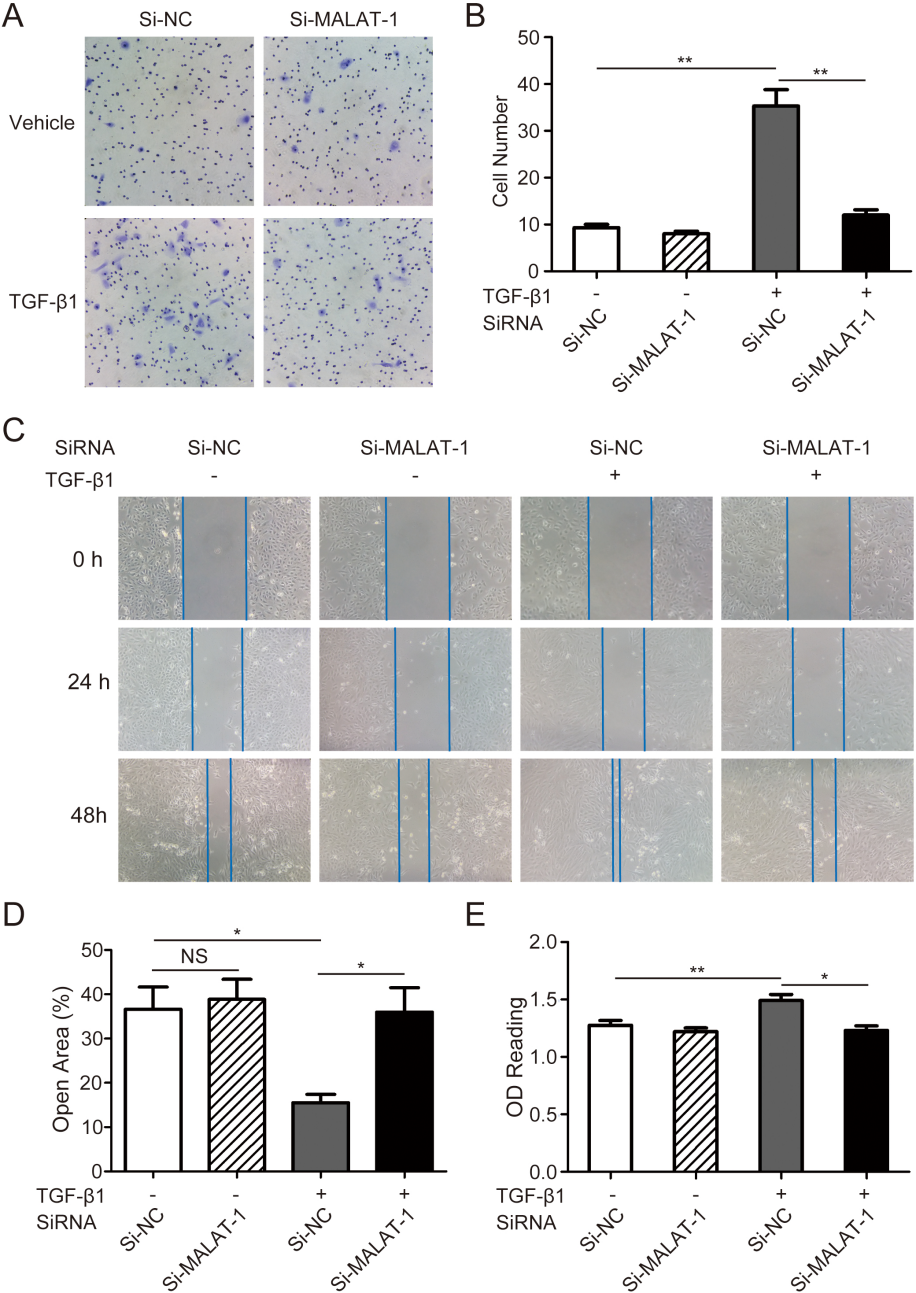
Downregulation of MALAT1 inhibited TGF-β1 induced migration and proliferation in RPE cells. A. ARPE-19 cells were transfected with MALAT1 SiRNA (Si-MALAT1) or negative control SiRNA (Si-NC) and were treated with or without TGF-β1 (10ng/ml) for 48 h. Cells were then subjected to transwell migration assay. B. The number of migrated cells was quantified by counting 5 random vision fields in a microscope (magnification: ×200). C. ARPE-19 cells were transfected with Si-MALAT1 or Si-NC. A scratch was then made to the cell monolayer and TGF-β1 (10ng/ml) was applied. Photographs were taken at indicated times. D. The ratios of remaining of gap at 48 h were calculated. E. After siRNA transfection and TGF-β1 incubation, cell proliferation was assessed using an MTT cell proliferation assay kit.

We then explored the role of MALAT1 on mobility and growth of RPE cells. Both the transwell and the scratch assay showed that down-regulation of MALAT1 significantly hamper the migration of RPE cells (Fig 4A–4D). The MTT assay clear showed that knockdown of MALAT1 by siRNA significantly inhibits the proliferation of ARPE-19 cells after 48 h treated with TGF-β1 (Fig 4E).

### MALAT1 activates the canonical Smad2/3 signaling of TGF-β

**Fig 5:**
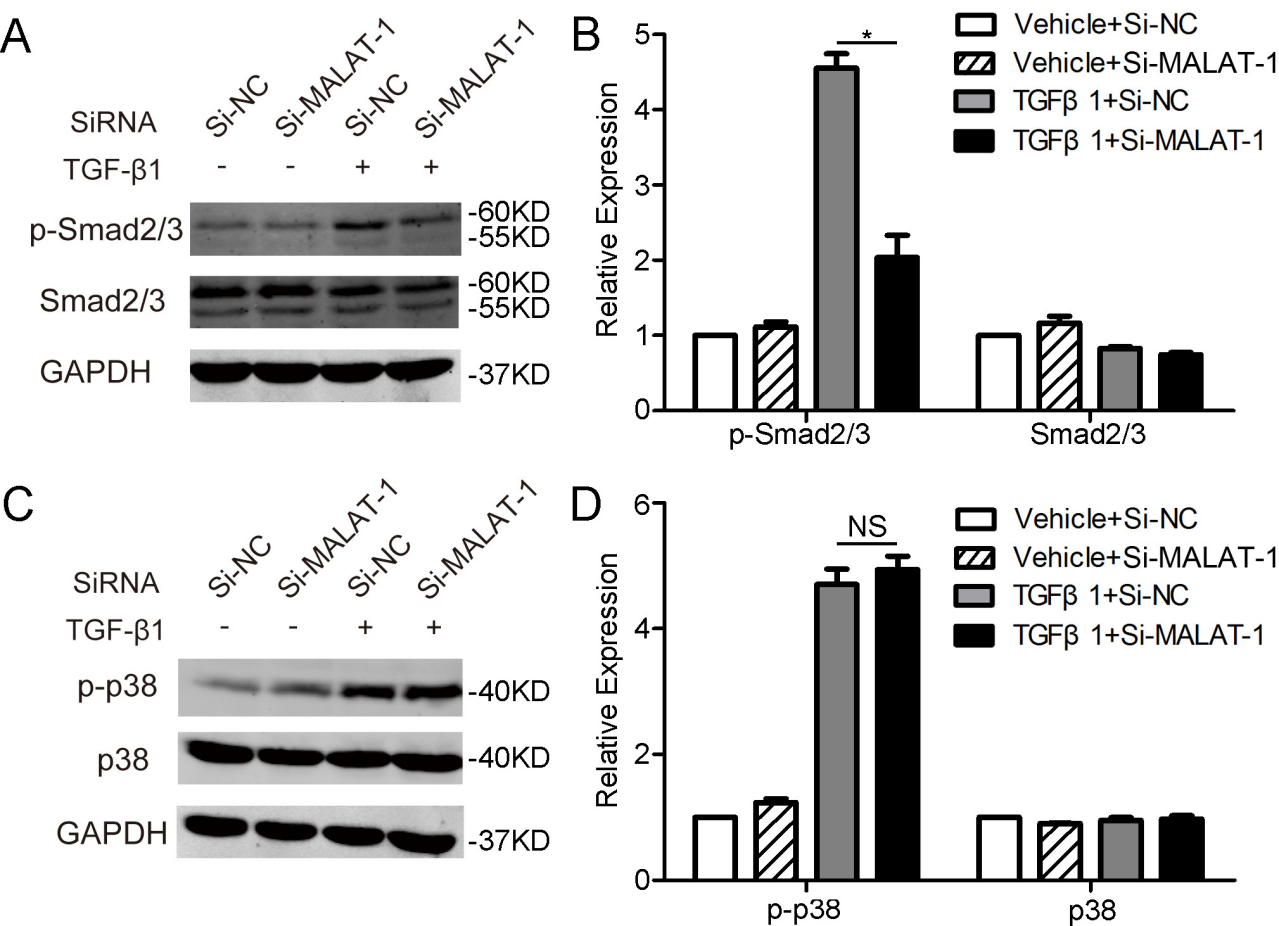
Downregulating MALAT1 inhibits the phosphorylation of Smad2/3. ARPE-19 cells transfected with Si-MALAT1 or Si-NC were treated with TGF-β1 (10 ng/ml) for 1 h. The expression of p-Smad2/3 and Smad2/3 (A), as well as p-38 and p38 (C) were examined by western blot. The relative protein expressions were quantified by normalizing to the GAPDH expression (B and D).

We then examined the effects of modulating MALAT1 expression on TGF-β signaling. Thus, the phosphorylation of Smad2/3 and p38, two well-known signaling pathways involved in TGF-β, were detected by western blot. It was shown that knockdown of MALAT1 dramatically inhibits TGF-β1 induced phosphorylation of Smad2/3, but has no obvious effect on phosphorylation of p38 (Fig 5A–5D). These results indicated that MALAT1-mediated TGF-β1-inuced EMT of RPE cells is, at least partially, through activating Smad2/3 signaling pathway.

### The expression of MALAT1 is increased in human primary RPE cells incubated with PVR vitreous

**Fig 6:**
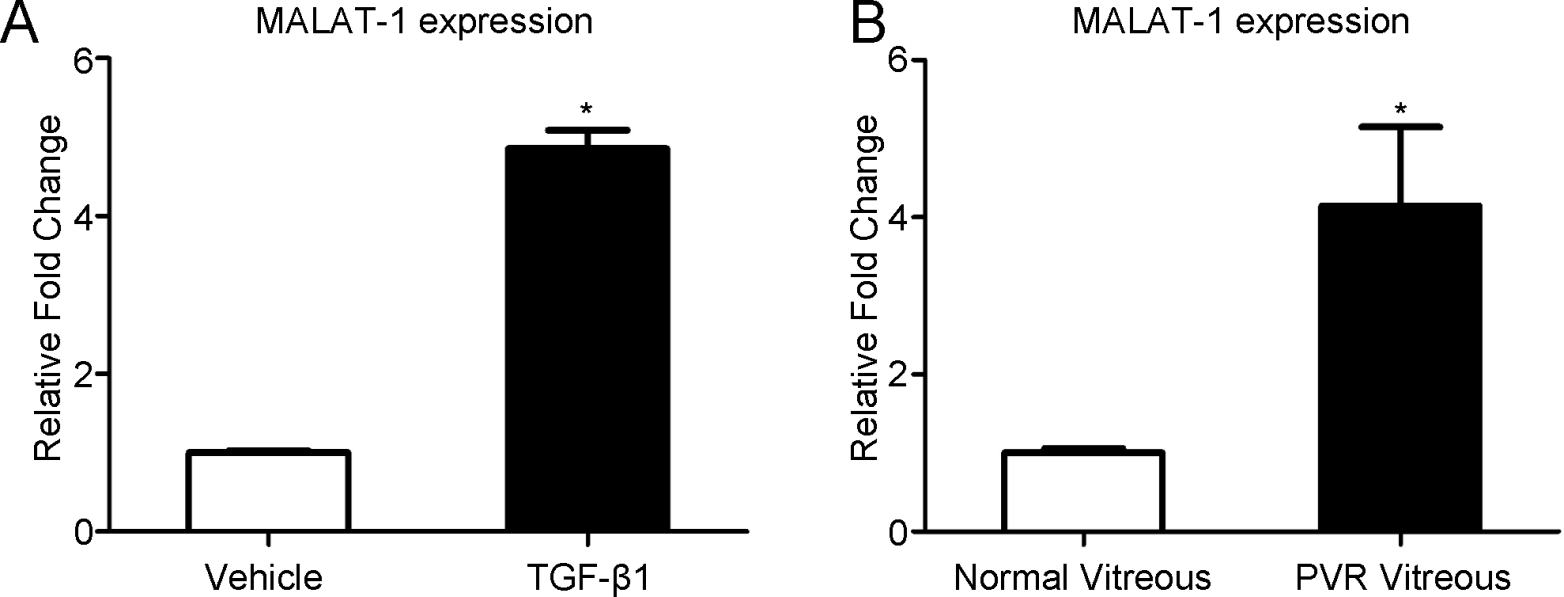
MALAT1 expression is increased in human primary RPE cells incubated TGF-β1 (10 ng/ml) (A) or PVR vitreous (B) as detected by RT-PCR.

To assess whether MALAT1 is involved in the pathological progress of PVR, we detected the expression of MALAT1 in human primary RPE cells treated with PVR vitreous samples. As TGF-β signaling is one of the most important pathway in the pathogenesis of PVR [4], we first stimulate human primary RPE cells with TGF-β1 (10ng/ml) for 48 h. Consistent with it is in ARPE-19 cells, TGF-β1 significantly increases the expression of MALAT1 in human primary RPE cells (Fig 6A). We then treated the cells with 4× diluted clinical PVR vitreous samples for 48 h. Cells treated with 4× diluted vitreous from normal donor eyes were set as control. The expression of MALAT1 is also up-regulated in human primary RPE cells treated with PVR vitreous (Fig 6B).

## Discussion

EMT is a physiological or pathological process that a fully polarized cell, which normally anchores to the basement membrane via its basal surface, undergoes multiple morphological and functional changes, and thereby acquires mesenchymal phenotypes including appearance of an elongated spindle-like morphology, enhanced migratory and invasive capacity, increased resistance to apoptosis, and prominently increased production of ECM components [22,23]. EMT is a hallmark in embryonic development, tumor metastasis, and organ fibrosis. Recent studies revealed that EMT of RPE cells is the main contributor to the pathogenesis of PVR, an ocular fibrotic disease characterized by the formation of contractile epiretinal membranes [4,7,24–26]. However, the underlying mechanism of EMT in RPE cells during the progression of PVR is far from clear. MALAT1 is a lncRNA required for the EMT process of cancer cells [19,20]. In this present study, for the first time, we confirmed that MALAT1 promotes EMT in RPE cells, which implies that MALAT1 may play a significant role in fibrotic diseases.

MALAT1 is firstly identified as a bio-marker for early stage non-small cell lung cancer. Later on, researchers found that MALAT1 is also highly expressed in diverse normal organs [27]. Moreover, during the pathogenesis and metastasis of tumor, MALAT1 is up-regulated and promotes the EMT, proliferation, and migration of tumor cells [19,20,28]. Fan Y et al. reported that administration of TGF-β up-regulates the expression of MALAT1 in bladder cancer cells [19]. Consistently, our present study found that the expression of MALAT1 is increased in RPE cells stimulated with TGF-β1 and reaches the apex at 48 h. It indicated that MALAT1 may play an essential role in the TGF-β induced activation of RPE cells, a hallmark in the development of PVR. Thus, we further explored the role of MALAT1 in RPE cells by knocking down MALAT1 using siRNA. In consistent with previous studies [19], knockdown of MALAT1 significantly inhibited the TGF-β1 induced EMT phenotypes, including the morphological change, up-regulation of α-SMA, fibronectin, and down-regulation of ZO-1 and E-Cadherin.

TGFβ-induced EMT acts through various signaling pathways including the canonical Smad2/3, and non-canonical p38, AKT, ERK1/2, and so on [7]. Here, we detected the effects of MALAT1 inhibition on two most well-known TGFβ signalings, Smad2/3 and p38. We found that MALAT1 acts through Smad2/3 rather than p38. Further studies are still needed to address the role of MALAT1 in other TGFβ signalings. Our previous study indicated that transcription factor Snail is involved in the TGF-β1 induced EMT in RPE cells [8]. Recently, Fan Y et al. revealed that in bladder cancer cells, MALAT1 induces EMT by ssociating with Suz12, a H3K27 methyltransferase required for the suppression of E-Cadherin expression by Snail [19,29]. Thus, combined with our present results, MALAT1 may act through Suz12-Snail to mediate the TGF-β1 induced EMT in RPE cells.

Uncontrolled migration and proliferation of RPE cells, partially as a result of EMT, are also hallmarks for the formation of pathological epiretinal membrane [4]. Recent studies indicated that the up-regulated MALAT1 is essential for the proliferation and migration in diverse cancer cells [30–32]. Our study also proved that knockdown of MALAT1 considerably suppresses the migration and proliferation of ARPE-19 cells treated with TGF-β1.

In the setting of PVR, as a result of blood-retinal barriers damage, the vitreous is rich of cytokines and growth factors. RPE cells exposed to the cytokine and growth factor-rich vitreous were then activated and eventually participated in the formation of the epiretinal membranes. However, normal vitreous samples do not contain much amount of cytokines and growth factors as the blood-retinal barrier is intact. Therefore, to further explore the potential role of MALAT1 in PVR, we simulated the pathological condition by treating the human primary RPE cells with PVR vitreous samples. Our data showed that, compared with normal vitreous, PVR vitreous treatment significantly increases the expression of MALAT1 in human primary RPE cells. We also confirmed that TGF-β1 is capable of up-regulating MALAT1 in primary RPE cells.

In conclusion, our results confirmed that MALAT1 is a critical mediator in the EMT, migration, and proliferation of RPE cells. MALAT1 might play a significant role in the pathogenesis of PVR. The role of MALAT1 in activating RPE cells opens new windows for understanding the mechanisms of PVR and may provide new potential therapeutic target.

## Supporting Information

S1 Table Primers sequences used in this study. (DOCX)

## References

[1] Kim IK, Arroyo JG (2002) Mechanisms in proliferative vitreoretinopathy. Ophthalmol Clin North Am 15: 81–86. 12064085 10.1016/s0896-1549(01)00008-6

[2] Cardillo JA, Stout JT, LaBree L, Azen SP, Omphroy L, Cui JZ, et al (1997) Post-traumatic Proliferative Vitreoretinopathy: The Epidemiologfic Profile, Onset, Risk Factors, and Visual Outcome. Ophthalmology 104: 1166–1173. 9224471 10.1016/s0161-6420(97)30167-5

[3] Sadaka A, Giuliari GP (2012) Proliferative vitreoretinopathy: current and emerging treatments. Clinical ophthalmology (Auckland, NZ) 6: 1325. 10.2147/OPTH.S27896 PMC3429288 22942638

[4] Pennock S, Haddock LJ, Eliott D, Mukai S, Kazlauskas A (2014) Is neutralizing vitreal growth factors a viable strategy to prevent proliferative vitreoretinopathy? Prog Retin Eye Res 40: 16–34. 10.1016/j.preteyeres.2013.12.006 24412519

[5] Machemer R, van Horn D, Aaberg TM (1978) Pigment epithelial proliferation in human retinal detachment with massive periretinal proliferation. American journal of ophthalmology 85: 181–191. 623188 10.1016/s0002-9394(14)75946-x

[6] Lei H, Rheaume MA, Kazlauskas A (2010) Recent developments in our understanding of how platelet-derived growth factor (PDGF) and its receptors contribute to proliferative vitreoretinopathy. Exp Eye Res 90: 376–381. 10.1016/j.exer.2009.11.003 19931527 PMC2824005

[7] Yang S, Li H, Li M, Wang F (2015) Mechanisms of epithelial-mesenchymal transition in proliferative vitreoretinopathy. Discov Med 20: 207–217. 26562474

[8] Li H, Wang H, Wang F, Gu Q, Xu X (2011) Snail involves in the transforming growth factor beta1-mediated epithelial-mesenchymal transition of retinal pigment epithelial cells. PLoS One 6: e23322 10.1371/journal.pone.0023322 21853110 PMC3154444

[9] Bastiaans J, van Meurs JC, van Holten-Neelen C, Nagtzaam NM, van Hagen PM, Chambers RC, et al (2013) Thrombin induces epithelial-mesenchymal transition and collagen production by retinal pigment epithelial cells via autocrine PDGF-receptor signaling. Invest Ophthalmol Vis Sci 54: 8306–8314. 10.1167/iovs.13-12383 24302586

[10] Li M, Li H, Liu X, Xu D, Wang F (2014) MicroRNA-29b regulates TGF-beta1-mediated epithelial-mesenchymal transition of retinal pigment epithelial cells by targeting AKT2. Exp Cell Res. 10.1016/j.yexcr.2014.09.026 25263462

[11] Chen X, Xiao W, Wang W, Luo L, Ye S, Liu Y. (2014) The complex interplay between ERK1/2, TGFbeta/Smad, and Jagged/Notch signaling pathways in the regulation of epithelial-mesenchymal transition in retinal pigment epithelium cells. PLoS One 9: e96365 10.1371/journal.pone.0096365 24788939 PMC4008562

[12] Dvashi Z, Goldberg M, Adir O, Shapira M, Pollack A (2015) TGF-beta1 Induced Transdifferentiation of RPE Cells is Mediated by TAK1. PLoS One 10: e0122229 10.1371/journal.pone.0122229 25849436 PMC4388737

[13] Chen X, Ye S, Xiao W, Luo L, Liu Y (2014) Differentially expressed microRNAs in TGFbeta2-induced epithelial-mesenchymal transition in retinal pigment epithelium cells. Int J Mol Med 33: 1195–1200. 10.3892/ijmm.2014.1688 24604358

[14] Mony S, Lee SJ, Harper JF, Barwe SP, Langhans SA (2013) Regulation of Na,K-ATPase beta1-subunit in TGF-beta2-mediated epithelial-to-mesenchymal transition in human retinal pigmented epithelial cells. Exp Eye Res 115: 113–122. 10.1016/j.exer.2013.06.007 23810808 PMC3796007

[15] Hatanaka H, Koizumi N, Okumura N, Kay EP, Mizuhara E, Hamuro J, et al (2012) Epithelial-mesenchymal transition-like phenotypic changes of retinal pigment epithelium induced by TGF-beta are prevented by PPAR-gamma agonists. Invest Ophthalmol Vis Sci 53: 6955–6963. 10.1167/iovs.12-10488 22956604

[16] Gamulescu MA, Chen Y, He S, Spee C, Jin M, Ryan SJ, et al (2006) Transforming growth factor beta2-induced myofibroblastic differentiation of human retinal pigment epithelial cells: regulation by extracellular matrix proteins and hepatocyte growth factor. Exp Eye Res 83: 212–222. 16563380 10.1016/j.exer.2005.12.007

[17] Yuan JH, Yang F, Wang F, Ma JZ, Guo YJ, Tao QF, et al (2014) A long noncoding RNA activated by TGF-beta promotes the invasion-metastasis cascade in hepatocellular carcinoma. Cancer Cell 25: 666–681. 10.1016/j.ccr.2014.03.010 24768205

[18] Matouk IJ, Raveh E, Abu-lail R, Mezan S, Gilon M, Gershtain E, et al (2014) Oncofetal H19 RNA promotes tumor metastasis. Biochim Biophys Acta 1843: 1414–1426. 10.1016/j.bbamcr.2014.03.023 24703882

[19] Fan Y, Shen B, Tan M, Mu X, Qin Y, Zhang F, et al (2014) TGF-beta-induced upregulation of malat1 promotes bladder cancer metastasis by associating with suz12. Clin Cancer Res 20: 1531–1541. 10.1158/1078-0432.CCR-13-1455 24449823

[20] Ying L, Chen Q, Wang Y, Zhou Z, Huang Y, Qiu F (2012) Upregulated MALAT-1 contributes to bladder cancer cell migration by inducing epithelial-to-mesenchymal transition. Mol Biosyst 8: 2289–2294. 22722759 10.1039/c2mb25070e

[21] Li H, Li M, Xu D, Zhao C, Liu G, Wang F (2014) Overexpression of Snail in retinal pigment epithelial triggered epithelial-mesenchymal transition. Biochem Biophys Res Commun 446: 347–351. 10.1016/j.bbrc.2014.02.119 24607896

[22] Lamouille S, Xu J, Derynck R (2014) Molecular mechanisms of epithelial-mesenchymal transition. Nat Rev Mol Cell Biol 15: 178–196. 10.1038/nrm3758 24556840 PMC4240281

[23] Thiery JP, Acloque H, Huang RY, Nieto MA (2009) Epithelial-mesenchymal transitions in development and disease. Cell 139: 871–890. 10.1016/j.cell.2009.11.007 19945376

[24] Umazume K, Barak Y, McDonald K, Liu L, Kaplan HJ, Tamiya S (2012) Proliferative vitreoretinopathy in the Swine-a new model. Invest Ophthalmol Vis Sci 53: 4910–4916. 10.1167/iovs.12-9768 22729438

[25] Feist RM Jr., King JL, Morris R, Witherspoon CD, Guidry C (2014) Myofibroblast and extracellular matrix origins in proliferative vitreoretinopathy. Graefes Arch Clin Exp Ophthalmol 252: 347–357. 10.1007/s00417-013-2531-0 24276562

[26] Casaroli-Marano RP, Pagan R, Vilaro S (1999) Epithelial-mesenchymal transition in proliferative vitreoretinopathy: intermediate filament protein expression in retinal pigment epithelial cells. Invest Ophthalmol Vis Sci 40: 2062–2072. 10440262

[27] Gutschner T, Hammerle M, Diederichs S (2013) MALAT1—a paradigm for long noncoding RNA function in cancer. J Mol Med (Berl) 91: 791–801. 23529762 10.1007/s00109-013-1028-y

[28] Zhou X, Liu S, Cai G, Kong L, Zhang T, Ren Y, et al (2015) Long Non Coding RNA MALAT1 Promotes Tumor Growth and Metastasis by inducing Epithelial-Mesenchymal Transition in Oral Squamous Cell Carcinoma. Sci Rep 5: 15972 10.1038/srep15972 26522444 PMC4629155

[29] Herranz N, Pasini D, Diaz VM, Franci C, Gutierrez A, Dave N, et al (2008) Polycomb complex 2 is required for E-cadherin repression by the Snail1 transcription factor. Mol Cell Biol 28: 4772–4781. 10.1128/MCB.00323-08 18519590 PMC2493371

[30] Ren S, Liu Y, Xu W, Sun Y, Lu J, Wang F, et al (2013) Long noncoding RNA MALAT-1 is a new potential therapeutic target for castration resistant prostate cancer. J Urol 190: 2278–2287. 10.1016/j.juro.2013.07.001 23845456

[31] Xu C, Yang M, Tian J, Wang X, Li Z (2011) MALAT-1: a long non-coding RNA and its important 3' end functional motif in colorectal cancer metastasis. Int J Oncol 39: 169–175. 10.3892/ijo.2011.1007 21503572

[32] Schmidt LH, Spieker T, Koschmieder S, Schaffers S, Humberg J, Jungen D, et al (2011) The long noncoding MALAT-1 RNA indicates a poor prognosis in non-small cell lung cancer and induces migration and tumor growth. J Thorac Oncol 6: 1984–1992. 10.1097/JTO.0b013e3182307eac 22088988

