# Supplementary material for: Long Non-Coding RNA MALAT1 Mediates Transforming Growth Factor Beta1-Induced Epithelial-Mesenchymal Transition of Retinal Pigment Epithelial Cells
Source: PLoS One. 2016 Mar 28;11(3):e0152687. doi: 10.1371/journal.pone.0152687 (PMC4809592; doi:10.1371/journal.pone.0152687)
Supplement: S1 Table — (DOCX) [file pone.0152687.s001.docx]

## S1 Table. Primers sequences used in this study

| Primers | Forward primer | Reverse primer | Product size |
| --- | --- | --- | --- |
| MALAT1 | GATTGAGGAGGCTGTGCTGT | CAGCTGCCTGCTGTTTTCTG | 112 |
| E-Cadherin | TCACGCTGTGTCATCCAACGG | TAGGTGTTCACATCATCGTCCGC | 192 |
| ZO-1 | AGCCATTCCCGAAGGAGTTGAG | ATCACAGTGTGGTAAGCGCAGC | 175 |
| αSMA | CAGAAGGAGATCACGGCCCTAG | CGGCTTCATCGTATTCCTGTTTG | 157 |
| Fibronectin | AAGACCATACCCGCCGAATG | GGCATTTGGATTGAGTCCCG | 109 |
| Snail | TTTCTGGTTCTGTGTCCTCT | TGTCAGCCTTTGTCCTGT | 179 |
| SLUG | ATGTCGGTTGTCTGGTTG | TCTCCTGTGTTTTGTTCTTGT | 115 |
| ZEB1 | AGTGTTTGTGATTGTGTTTGA | GATGAAGGCGGGTTAGAG | 141 |
| GAPDH | CATCAGCAATGCCTCCTGCAC | TGAGTCCTTCCACGATACCAAAGTT | 86 |
